# A Case of Hypercalcaemia With Suppressed Parathyroid Hormone in a Middle-Aged Female: Diagnostic Challenge and Association With Hyperthyroidism

**DOI:** 10.7759/cureus.72020

**Published:** 2024-10-21

**Authors:** Bharathiraman Chengalpet Jaishankar, Saifuddin Mohammad Kibria, Aditya Sudarshanan, Niharika Patlolla, Cornelius Fernandez James

**Affiliations:** 1 Internal Medicine, Pilgrim Hospital, United Lincolnshire Hospitals NHS Trust, Boston, GBR; 2 Acute Medicine, Pilgrim Hospital, United Lincolnshire Hospitals NHS Trust, Boston, GBR; 3 Endocrinology and Metabolism, Pilgrim Hospital, United Lincolnshire Hospitals NHS Trust, Boston, GBR

**Keywords:** autoimmune, hypercalcaemia, hyperparathyroidism, hyperthyroidism, malignancy

## Abstract

Hypercalcaemia with suppressed parathyroid hormone (PTH) typically raises concern for malignancy-related, granulomatous disorder-related or drug-related hypercalcaemia but can occasionally be caused by less common conditions. We present the case of a middle-aged female with hypercalcaemia with reduced PTH levels despite vitamin insufficiency, who was eventually diagnosed with autoimmune hyperthyroidism. The diagnostic challenge arose from the atypical association of hyperthyroidism and hypercalcaemia. This case highlights the importance of considering hyperthyroidism in the differential diagnosis of hypercalcaemia.

## Introduction

Hypercalcaemia, defined as elevated serum calcium levels, often prompts an evaluation of parathyroid hormone (PTH) levels to differentiate between parathyroid-related and non-parathyroid-related hypercalcaemia. In cases of hypercalcaemia with suppressed PTH, clinicians often consider malignancy, granulomatous disorders, drugs and other systemic conditions. However, hyperthyroidism is a lesser-known cause of hypercalcaemia and can pose a diagnostic challenge. This case report describes a patient presenting with symptomatic hypercalcaemia with suppressed PTH, in whom the simultaneous workup showed the presence of hyperthyroidism, leading to an eventual diagnosis of autoimmune hyperthyroidism-mediated hypercalcaemia.

## Case presentation

A 53-year-old female presented to her general practitioner (GP) surgery for routine follow-up after a recent total hip replacement surgery. She had no significant past medical or family history and was only on vitamin D supplements. She has not had any immobilisation after the hip surgery. During the consultation, she reported significant constipation but did not report any other related symptoms such as abdominal pain, vomiting, loss of appetite or weight loss. Blood investigations revealed that she had moderate hypercalcaemia with an adjusted calcium of 3.06 mmol/L and phosphate of 1.25 mmol/L. Subsequently, PTH and 25-hydroxy vitamin D levels were requested, which revealed suppressed PTH level (0.8 pmol/L) alongside vitamin D insufficiency (39 nmol/L). Hypercalcaemia with suppressed PTH suggested a non-parathyroid cause of hypercalcaemia.

Thyroid function tests, which were requested to evaluate constipation, revealed a suppressed thyroid-stimulating hormone (TSH: <0.01 mU/L) with raised free thyroxine (T4) levels (43.6 pmol/L) indicating primary overt hyperthyroidism.

The GP subsequently sought advice and guidance from endocrinology. Given the clinical suspicion of hyperthyroidism as the likely cause of hypercalcaemia, they recommended initiating treatment with carbimazole 15 mg once a day with serial thyroid function test, calcium and PTH monitoring. The endocrine team reassured the GP surgery and advised to avoid investigating for other non-parathyroid causes of hypercalcaemia including malignancy.

**Figure 1:**
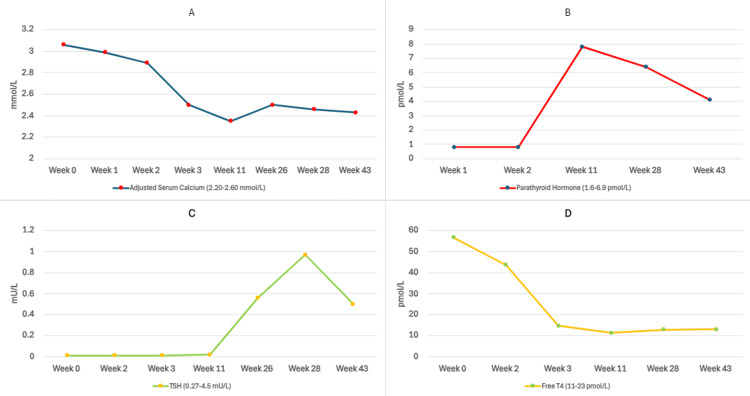
Trends in the blood investigations from week 0 to week 43 (A) Adjusted serum calcium levels, (B) PTH levels, (C) TSH levels and (D) free T4 levels TSH, thyroid-stimulating hormone; T4, thyroxine; PTH, parathyroid hormone

In one week, the carbimazole dose was up-titrated to 30 mg daily. This has resulted in the normalisation of free T4 levels (14.6 pmol/L), as well as adjusted calcium levels (2.50 mmol/L) by week 4, thereby relieving constipation, although her TSH levels had remained suppressed (0.02 mU/L) (Table 1 and Figure 1). The normalisation of hypercalcaemia with the achievement of euthyroidism solidified the argument of hyperthyroidism-induced hypercalcaemia. Further, the patient later tested positive for TSH receptor antibodies (TRAb), confirming the diagnosis of autoimmune hyperthyroidism. Ten months after the diagnosis, blood tests revealed euthyroid status with normal PTH and calcium while on carbimazole 5 mg once daily and cholecalciferol 800 units once daily (Table 1 and Figure 1).

**Table 1:** Blood investigations TSH, thyroid-stimulating hormone; T4, thyroxine

Investigations	Units	Reference range	Week 0	Week 1	Week 2	Week 3	Week 11	Week 26	Week 28	Week 43
Serum calcium (adjusted)	mmol/L	2.20-2.60	3.06	2.99	2.89	2.5	2.35	2.5	2.46	2.43
Phosphate	mmol/L	0.80-1.50	1.25	1.32	1.22	-	1.14	1.18	1.3	1.2
Alkaline phosphatase	U/L	30-130	-	-	67	-	190	136	134	92
Parathyroid hormone	pmol/L	1.6-6.9	-	0.8	0.8	-	7.8	-	6.4	4.1
TSH	mU/L	0.27-4.5	0.01	-	0.01	0.01	0.02	0.56	0.97	0.5
Free T4	pmol/L	11-23	56.8	-	43.6	14.6	11.3	-	12.9	13

## Discussion

Most of the cases that we encounter with hypercalcaemia are due to either primary hyperparathyroidism or malignancy, which in combination contributes to over 90% of hypercalcaemia cases [1,2]. While primary hyperparathyroidism is the most likely cause when you are dealing with raised PTH levels (Table 2), a suppressed PTH level warrants a prompt workup for malignancy based on the clinical presentation [1,3]. In this case, despite PTH levels being suppressed, we decided against the latter as an alternative explanation for ‘hypercalcaemia with suppressed PTH’ in the form of hyperthyroidism was evident.

**Table 2:** Causes of PTH-dependent hypercalcaemia Source: [4] PHPT, primary hyperparathyroidism; MEN1, multiple endocrine neoplasia type 1; MEN2A, multiple endocrine neoplasia type 2A; MEN4, multiple endocrine neoplasia type 4; PTH, parathyroid hormone

Pathology	Causes
Primary hyperparathyroidism, sporadic	Adenoma, hyperplasia and carcinoma
Primary hyperparathyroidism, familial	MEN1, MEN2A, MEN4, hyperparathyroidism-jaw tumour syndrome and familial isolated PHPT
Familial benign hypercalcaemia	Familial hypocalciuric hypercalcaemia
Tertiary hyperparathyroidism	Chronic kidney disease and phosphate therapy in hypophosphatemia rickets
Medication-induced	Lithium and thiazides

Another important cause we must consider is vitamin D-mediated hypercalcaemia. This is the most common drug-induced cause of hypercalcaemia [5]. The clinical presentation would echo those of any other cause of hypercalcaemia, including neuropsychiatric, gastrointestinal, cardiovascular and renal manifestations [6]. Apart from vitamin D, the two other drugs that we must consider are lithium and thiazides, as they frequently cause drug-induced hypercalcaemia, albeit with increased PTH levels [5].

Once the above-mentioned common causes of hypercalcaemia are ruled out, we must explore the likelihood of uncommon causes of hypercalcaemia including hyperthyroidism, adrenal insufficiency, granulomatous diseases, immobility, lymphoma and myeloma (Table 3). In hyperthyroidism, calcium levels are usually expected to be only mildly elevated (2.6-3.0 mmol/L) [7], but as seen in our case, there have been a few instances where it has been presented with moderate to severe hypercalcaemia [8,9]. Only about 20% of the patients with hyperthyroidism present with hypercalcaemia. Most of these patients, in addition to hypercalcaemia with suppressed PTH, would have classical hyperthyroidism symptoms including palpitation, shakiness, irritable mood and weight loss [9].

**Table 3:** Causes of PTH-independent hypercalcaemia Source: [4] CMV, cytomegalovirus; PTH, parathyroid hormone

Pathology	Causes
Malignancy-associated	Humoral hypercalcaemia of malignancy, local osteolytic hypercalcaemia and extra-renal calcitriol-mediated hypercalcaemia
Granulomatous disorders	Sarcoidosis, Wegener’s granulomatosis, tuberculosis, histoplasmosis and berylliosis
Medication-induced	Vitamin D, lithium, thiazides, vitamin A, milk-alkali syndrome, aluminium intoxication, aminophylline/theophylline and tamoxifen
Endocrine disorders	Hyperthyroidism, hypoadrenalism, pheochromocytoma, VIPoma and Jansen’s metaphyseal chondrodysplasia
Immobilisation	-
Viral infections	HIV and CMV

Hypercalcaemia occurs in 6.5%-8.4% of patients with adrenal insufficiency, with Addison’s disease accounting for 5.5%-6.0% of these cases, making hypercalcaemia more prevalent in Addison’s disease compared to other forms of adrenal insufficiency [10,11]. Graves’ disease and Addison’s disease can coexist in autoimmune polyglandular syndrome type 2 [12].

The pathophysiology behind hyperthyroidism-induced hypercalcaemia is still not completely understood. When long bones of laboratory animals were cultured in thyroid hormone, there was a marked increase in osteoclastic activity with subsequent release of calcium [13]. Earlier studies have suggested enhanced sensitivity of β-adrenergic receptors to catecholamines in hyperthyroidism. In addition to this, there is an increased sensitivity of the bone to PTH [14,15]. There is evidence to suggest that the receptor activator of nuclear factor κB ligand (RANKL) activity is increased due to the enhanced sensitivity of the bone to IL-6, enhancing osteoclastic activity [16]. Hyperthyroidism also increases bone turnover and mobilises calcium into the bloodstream from the bone. Moreover, the relative adrenal insufficiency that accompanies hyperthyroidism is well-known to cause hypercalcaemia [17].

When we have ascertained that hyperthyroidism is the cause of raised calcium, we should investigate further regarding the aetiology of hyperthyroidism, autoimmune (Graves’ disease) or toxic nodular disease. While the clinical pictures of acropachy, alopecia, thyroid eye disease, leucopenia, splenomegaly and thymic enlargement are more common in autoimmune hyperthyroidism, hypercalcaemia on its own is quite non-specific [18].

Hypercalcaemia manifests through a variety of systemic symptoms (Table 4), often categorised as ‘painful bones, abdominal groans, renal stones, moans, thrones and psychiatric overtones’. Gastrointestinal symptoms (‘groans’) include abdominal pain, nausea, vomiting and rare complications such as peptic ulcers or pancreatitis. Skeletal issues (‘bones’) encompass pain, osteoporosis, arthritis and fractures, while ‘stones’ refer to kidney stones causing discomfort. Fatigue and general malaise are described as ‘moans’. ‘Thrones’ pertain to increased urination and constipation, whereas ‘psychiatric overtones’ involve cognitive and emotional disturbances such as lethargy, confusion, depression and memory problems [19].

**Table 4:** Clinical presentation of hypercalcaemia

System	Clinical features
Skeletal	Bone pain, arthritis, fractures, osteopenia and osteoporosis
Abdomen	Nausea, vomiting, constipation, weight loss and peptic ulcer
Renal	Renal colic, renal impairment, haematuria, thirst, oliguria and polyuria
Neuromuscular/neuropsychiatric	Fatigue, lethargy, muscle weakness, impaired concentration, insomnia, confusion, depression, anxiety, irritability, anorexia, delirium and coma
Cardiovascular	Hypertension, short QT interval, J waves and ventricular arrhythmias

In this case, the patient presented with only constipation, lacking other hallmark symptoms of hypercalcaemia. Interestingly, despite having overt hyperthyroidism, the patient was clinically asymptomatic, which, although rare, has been observed in some hyperthyroid individuals [20].

**Figure 2:**
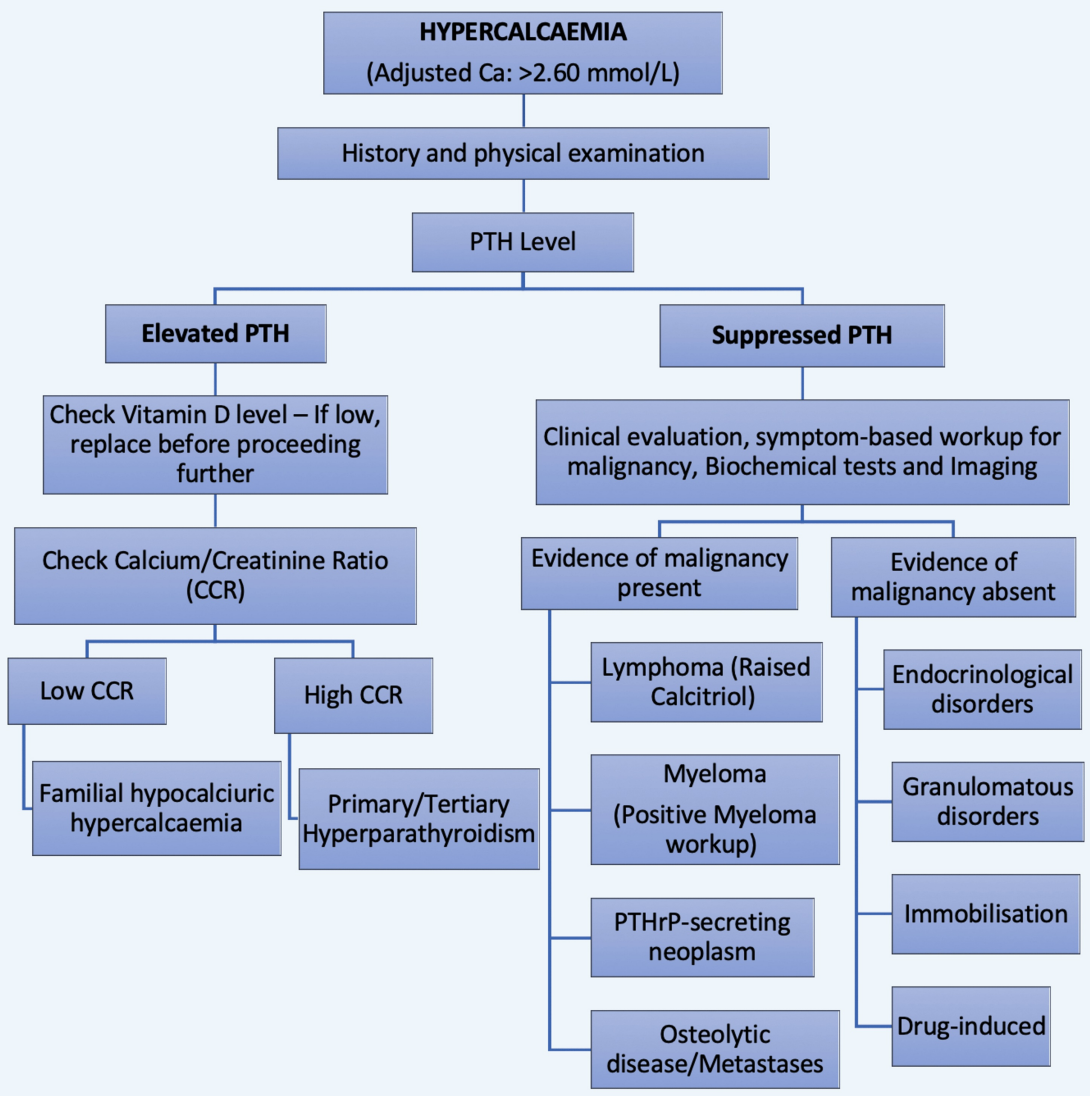
Diagnostic workup for hypercalcaemia Original figure PTH, parathyroid hormone; PTHrP, PTH-related peptide; Ca, calcium

The diagnostic workup for hypercalcaemia (Figure 2) begins with a thorough history, physical examination and measurement of serum parathyroid hormone (PTH) levels. If PTH is elevated, vitamin D levels are checked and, if low, replaced before proceeding with further evaluation. The calcium/creatinine ratio (CCR) is then assessed. Low CCR (<0.01) suggests familial hypocalciuric hypercalcaemia, while high CCR (>0.02) points towards primary or tertiary hyperparathyroidism. If PTH is suppressed, further clinical and biochemical evaluations, including imaging, are required to look for malignancy. If evidence of malignancy is found, potential causes include lymphoma, multiple myeloma, PTH-related peptide (PTHrP)-secreting neoplasms or osteolytic metastases. In the absence of malignancy, other causes such as endocrine disorders, granulomatous diseases, immobilisation or uncommon drug-induced hypercalcaemia should be considered.

Once properly diagnosed, the treatment remains straightforward with the initiation of anti-thyroid medication. The calcium levels are expected to be corrected with the return of the euthyroid state.

## Conclusions

This case illustrates symptomatic hypercalcaemia secondary to hyperthyroidism. The successful management of the thyroid disorder corrected the hypercalcaemia, confirming the causative relationship. Although malignancy is the most common cause of hypercalcaemia with suppressed PTH, a malignancy workup is not always necessary if an alternative diagnosis is strongly supported.
